# Priority effects of early successional insects influence late successional fungi in dead wood

**DOI:** 10.1002/ece3.1751

**Published:** 2015-10-12

**Authors:** Rannveig Margrete Jacobsen, Tone Birkemoe, Anne Sverdrup‐Thygeson

**Affiliations:** ^1^ Department of Ecology and Natural Resource ManagementNorwegian University of Life SciencesAasNorway

**Keywords:** Coarse woody debris, ecological engineer, facilitation, feeding guild, interaction, saproxylic, spore dispersal

## Abstract

Community assembly is an integral process in all ecosystems, producing patterns of species distributions, biodiversity, and ecosystem functioning. Environmental filters and colonization history govern the assembly process, but their relative importance varies depending on the study system. Dead wood decomposition is a slow process, allowing decomposer communities to develop within a slowly changing substrate for decades. Despite this, there are few long‐term studies of priority effects from colonization history in this ecosystem. In this study, we investigate the importance of insects in early succession of dead wood on the fungal community present one decade later. Sixty aspen trees were killed in two study landscapes, each tree producing one aspen high stump and log. Insects were sampled with flight interception traps during the first 4 years after tree death, and fungal fruiting bodies were registered in year twelve. We found positive priority effects of two fungivorous beetles, the sap beetle *Glischrochilus quadripunctatus* and the round fungus beetle *Agathidium nigripenne*, on the Artist's bracket (*Ganoderma applanatum*) and a positive priority effect of wood‐boring beetles on the ascomycete Yellow fairy cup (*Bisporella citrina*). The Aspen bracket (*Phellinus tremulae*) did not respond to insects in early succession of the dead wood. Our results suggest that early successional insects can have significant, long‐lasting effects on the late successional fungal community in dead wood. Also, the effect can be specific, with one fungus species depending on one or a few fungivorous beetle species. This has implications for decomposition and biodiversity in dead wood, as loss of early colonizing beetles may also affect the successional pathways they seem to initiate.

## Introduction

To understand patterns in species distributions, biodiversity, and ecosystem function, it is vital to understand the process of community assembly. Community assembly can be considered a purely deterministic process governed by abiotic factors such as nutrient availability and climate, or it can be influenced by colonization history (Drake 1991). Colonization history introduces a stochastic element which might lead to multiple stable states for similar habitats and thus increase biodiversity on a large scale (Chase 2010). There are several studies showing a marked effect of colonization history, often called a priority effect (Alford and Wilbur 1985; Shorrocks and Bingley 1994; Ejrnæs et al. 2006; Kennedy et al. 2009; Chase 2010; Dickie et al. 2012; Rasmussen et al. 2014). Short‐term experimental studies have shown that manipulating arrival order of species can strongly affect not only species composition and richness, but also ecosystem function (Fukami et al. 2010; Dickie et al. 2012). In most ecosystems, community assembly is probably affected by both abiotic factors and priority effects, but the relative importance varies (Chase 2003, 2010).

Depending on the system, colonization history might only influence the community initially (Cifuentes et al. 2010), or it can have more long‐lasting effects (Chase 2010; van de Voorde et al. 2011; Weslien et al. 2011). Priority effects of species arriving early can be positive or negative for the late successional species, leading to facilitative or inhibitory succession (Connell and Slatyer 1977). Connell and Slatyer (1977) suggested decomposer communities as a system where species assemblages could develop through facilitative succession. Their reasoning was that initial decomposition by early successional species might make the substrate more accessible for species in late succession. Whether facilitative or inhibitory, priority effects are likely to be strong in decomposer communities due to the changeable nature of the habitat.

The decomposer community associated with dead wood constitutes a major component of the biodiversity in boreal forests (Stokland et al. 2012), including a large number of endangered species (Gärdenfors 2010; Kålås et al. 2010; Rassi et al. 2010). The dead wood community is mainly composed of insects and fungi. Wood‐decay fungi have been shown to compete intensely for resources both in laboratory trials and in the field (Boddy 2000), and the competitive balance is influenced by the volume of wood each competitor controls (Holmer and Stenlid 1993). Furthermore, consistent patterns in fungal succession have been documented, with successor species following specific predecessor species (Niemelä et al. 1995; Ottosson et al. 2014). Thus, it is not surprising that strong priority effects have been found between wood‐decay fungi, affecting species richness and wood‐decay rate (Fukami et al. 2010; Dickie et al. 2012). This indirectly affects wood‐living insects, as several studies have shown a structuring effect of the fungal community on the species assemblage of wood‐living insects (Kaila et al. 1994; Jonsell et al. 2005; Abrahamsson et al. 2008; Leather et al. 2013). However, fungivorous insects can also affect fungal colonization history by acting as vectors for spores (Lim 1977; Tuno 1999; Persson et al. 2009; Strid et al. 2014) and may shift the competitive balance between fungi by preferential grazing (Crowther et al. 2011). Furthermore, wood‐boring insects can function as ecological engineers that alter the habitat by tunneling under the bark and into the wood, potentially affecting both insects and fungi (Buse et al. 2008; Weslien et al. 2011; Strid et al. 2014; Ulyshen 2014).

Current studies on the effect of insects on species composition of fungi mainly span a few years or less (Müller et al. 2002; Strid et al. 2014), while the process of decomposition and succession in dead wood can span decades (Mäkinen et al. 2006). In this study, we use a dataset spanning more than 10 years to investigate long‐term priority effects of beetles in early succession on wood‐decay fungi in late succession of aspen (*Populus tremula* L.) dead wood. To our knowledge, there has only been one previous study of long‐term priority effects in dead wood communities (Weslien et al. 2011). Weslien et al. (2011) showed that early colonizing wood‐boring beetles (Coleoptera) affect subsequent establishment of the common wood‐decay fungus the Red‐belt conk (*Fomitopsis pinicola* (Sw.: Fr.) P. Karst.) in dead wood of spruce (*Picea abies* (L.) H. Karst.). We advance upon this knowledge by studying three species of fungi with contrasting life‐history strategies and their response to not only wood‐boring beetles, but also fungivorous beetles. Furthermore, we include two different forest environments in our study design, which allows us to assess whether the priority effects are conditional upon surrounding environment. Thus, our study tests the generality of the hypothesis that beetles in early succession of dead wood exert priority effects on fungi in late succession.

## Materials and Methods

The field study was conducted in two landscapes in southern Norway, in the south boreal vegetation zone (Moen 1998), Losby forest holdings in Østmarka (Lat. 55.98, Long. 10.68, 150–300 masl) and Løvenskiold‐Vækerø forest holdings in Nordmarka (Lat. 54.49, Long. 21.24, 200–500 masl). Both forest holdings were managed as sustainable production forests within the regulations of the PEFC (the Programme for the Endorsement of Forest Certification schemes, Norway, pefcnorway.org). Both landscapes consisted of forest dominated by spruce (*Picea abies*), with pine (*Pinus sylvestris* L.), birch (*Betula pubescens* Ehrh.), and aspen (*Populus tremulae*) as subdominants.

In 2001, 60 study sites were chosen with a minimum distance of 100 m between the sites, each containing a mature aspen tree with diameter ≥20 cm at breast height (1.3 m above ground) (Sverdrup‐Thygeson and Birkemoe 2009). Within each study landscape, 15 study sites were established in closed canopy forest (aged 90–120 years) and 15 study sites in open, clear‐cut forest areas (2–4 years since clear‐cutting), each site being surrounded by a minimum of 10 m of the relevant habitat type. In the late fall of 2001, all 60 trees were cut at about 4 m above ground using detonating chord. Thus, after 2001, each site contained one aspen log and one aspen high stump.

**Figure 1:**
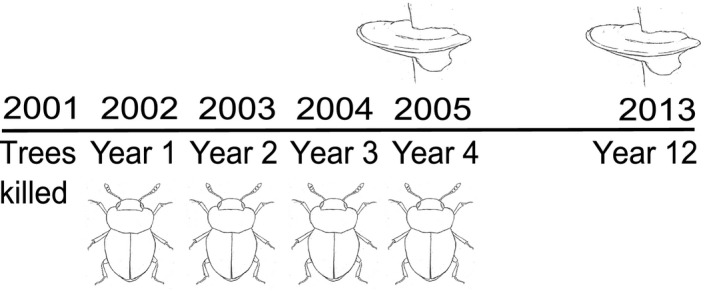
Time line showing time of tree death, followed by 4 years of insect sampling and registration of fungal fruiting bodies 4 years and 12 years after tree death.

In spring 2002, trunk window traps (40 cm × 60 cm) were mounted on the aspen high stumps, facing south and with the lower edge of the window pane 1 m above ground. The window traps collected insects by flight interception from medio May to medio August for 4 years following tree death, that is from 2002 to 2005 (Fig. 1). All beetle (Coleoptera) individuals were identified to species and categorized according to tree species preference and feeding guild according to the literature (Hansen et al. 1908–1965; Palm 1959; Hågvar 1999; Schigel 2011) and The Saproxylic Database compiled by Dahlberg and Stokland (2004) (accessible at http://radon.uio.no/WDD/Login.aspx?ReturnUrl=%2Fwdd%2FDefault.aspx). Vindstad and colleagues (unpublished data) are conducting a thorough analysis of the beetle communities for later publication. In the current paper, data from all 4 years of insect sampling were pooled in the statistical analysis.

In 2005, a precursory registration (presence/absence) of fungal fruiting bodies on high stumps and logs was conducted, identifying all polypores and a few other easily recognizable species. At the same time, proportion of bark left on the logs and high stumps was recorded.

In 2013, 12 years after tree death, fungal fruiting bodies of macrofungi on high stumps and logs, both Basidiomycetes and Ascomycetes, were registered (presence/absence) and identified to species. Only bark fungi that could be identified in the field were included. Fungi were categorized according to tree species preference recorded in the literature (Ryvarden and Melo 2014). High stumps and/or logs were missing at five sites in 2013, resulting in a total of 55 sites for analysis.

The fruiting body registration from 2013 was analyzed to explain distribution of certain fungus species, while the fruiting body registration from 2005 was only used to confirm whether these species had established at this point in succession and thus aid interpretation of the results.

Fruiting body surveys have certain methodological drawbacks, mainly the potential presence of a species as mycelium without fruiting body. However, high‐throughput sequencing of mycelium in dead wood has shown that well‐established species with high mycelial abundance tend to also have high fruiting rate (Ovaskainen et al. 2013). Thus, fruiting body surveys seem to be good indicators of dominating species.

Unless otherwise stated, all data were compiled to site level for analysis, combining fungal fruiting body registrations for high stumps and logs (presence at either high stump or log resulted in presence at site level).

### Study species

Only five species of wood‐decay fungi in late succession met the demands of occurrence at 10–45 sites (of 55) and preference for deciduous wood. Three of these species were chosen for their contrasting biology, in order to analyze for possible effect of early successional beetles; *Ganoderma applanatum* (Pers.) Pat.*, Phellinus tremulae* (Bondartsev) Bondartsev & B.N. Borisov, and *Bisporella citrina* (Batsch: Fr.) Korf & S.E. Carp. *B. citrina* is an annual ascomycete, while *G. applanatum* and *P. tremulae* are both basidiomycetes and perennial polypores. Furthermore, *P. tremulae* often parasitically infects living aspen trees (Ryvarden and Melo 2014), while both *G. applanatum* and *B. citrina* usually only colonize the trees after death.

Of the beetles collected in the first 4 years after tree death, species of two feeding guilds were used to explain occurrence patterns for the three species of fungi in year 12; fungivorous and wood‐boring beetles. Only species with a known affinity for dead wood of deciduous trees were included.

The fungivore guild included the fungivorous species in eight families: Ciidae, Endomychidae, Erotylidae, Latridiidae, Leiodidae, Ptinidae, Staphylinidae, and Nitidulidae. Interactions between fungivores and fungi depend on beetle feeding preferences, which can be species specific and are unknown for many species. Each species could not be tested separately, as that would lead to problems with multiple testing, so we initially tested for effect on the family level as a screening process for species‐specific effects. If there was a near significant effect of family (*P* < 0.1), the most abundant species in the families were also tested for effect in separate analyses. For most of the families, a few species accounted for almost all of the sampled individuals.

The wood‐borer guild included wood‐feeding species in three families: Cerambycidae, Curculionidae, and Ptinidae, the latter including only *Ptilinus fuscus* (Geoffroy, 1785). The wood‐borer guild was not partitioned further, as the hypothesized effect of wood borers as ecosystem engineers was expected to depend mostly on abundance of wood borers in general, and not on species‐specific traits other than guild membership. For abundance of wood borers sampled in the window traps to affect the fungi in the aspen dead wood through habitat alteration, abundance had to reflect use of the substrate. This connection was confirmed for a subset of species, including the numerically dominant wood‐borer *Rusticoclytus rusticus* (Linnaeus, 1785), in an earlier for study (Sverdrup‐Thygeson and Birkemoe 2009).

### Statistical methods

Generalized linear models (GLMs) with binomial distribution and logit link were used to test whether beetle abundance (fungivores or wood borers) affected the presence or absence of each of the three species of fungi. Wood‐boring beetles and each family of fungivores were tested separately. Habitat type (open or closed forest), site coordinates, and interaction between beetle abundance and habitat were included in all models, but the interaction was excluded if it was insignificant.

For fungivorous beetles significantly (*P* < 0.05) or near significantly (*P* < 0.10) associated with any of the three species of fungi, we also tested whether these fungivores were associated with the fungi registered in year 4 (with occurrences at 10–50 of 60 sites), to check whether the association with fungi in year 12 might be an indirect correlation due to attraction to fungi in year 4.

Effect of wood‐borer abundance on bark loss from logs and high stumps in early stages of decay was tested by a GLM with mean bark cover of the aspen dead wood at each site as response variable. For fungi responding to wood‐borer abundance*,* effect of bark cover in year 4 and dead wood object type (high stump or log) on occurrence of fruiting bodies in year 12 was also tested with GLMs. In these tests, the data for high stumps and logs were separated, resulting in two observations of all variables at most sites (*n* = 106).

All GLMs were evaluated with Pearson residual plots, Cooks distance, and the Hosmer–Lemeshow goodness of fit test (Hosmer and Lemeshow 2004). All analyses were conducted in R 3.1.1 (R Core Team 2014).

## Results

In total, 552 beetle species (19 512 individuals) were sampled during the first 4 years after tree death, of which 277 species (13 476 individuals) were wood‐living beetles associated with deciduous trees. The wood‐borer guild consisted of 23 species (961 individuals) and the fungivore guild of 56 species (3456 individuals) (Table S1). Both beetle guilds were significantly more abundant in open, clear‐cut forest than in the closed, mature forest (Fig. S1).

In the precursory registration of fungal fruiting bodies in year 4 after tree death, 14 species of fungi were registered. The most common species were *Trametes ochracea* (Pers.) Gilb. & Ryvarden (present at 50 of 60 sites) and *Chondrostereum purpureum* (Pers.: Fr.) Pouzar (present at 41 sites). Of the three fungus species from year 12 selected for analysis, only *P. tremulae* occurred already in year 4 (present at 24 of 60 sites).

In year 12 after tree death, 62 species of fungi were registered on the aspen high stumps and logs (including one species from the Norwegian Red List (Kålås et al. 2010), *Antrodia mellita* Niemelä & Penttilä). The most common species was *T. ochracea* which was present at 44 of 55 sites, followed by *B. citrina* at 41 sites (Table S2). *P. tremulae* was present at 19 sites and *G. applanatum* at 14 sites.

### Effects of early fungivorous beetles on late successional fungi

**Figure 2:**
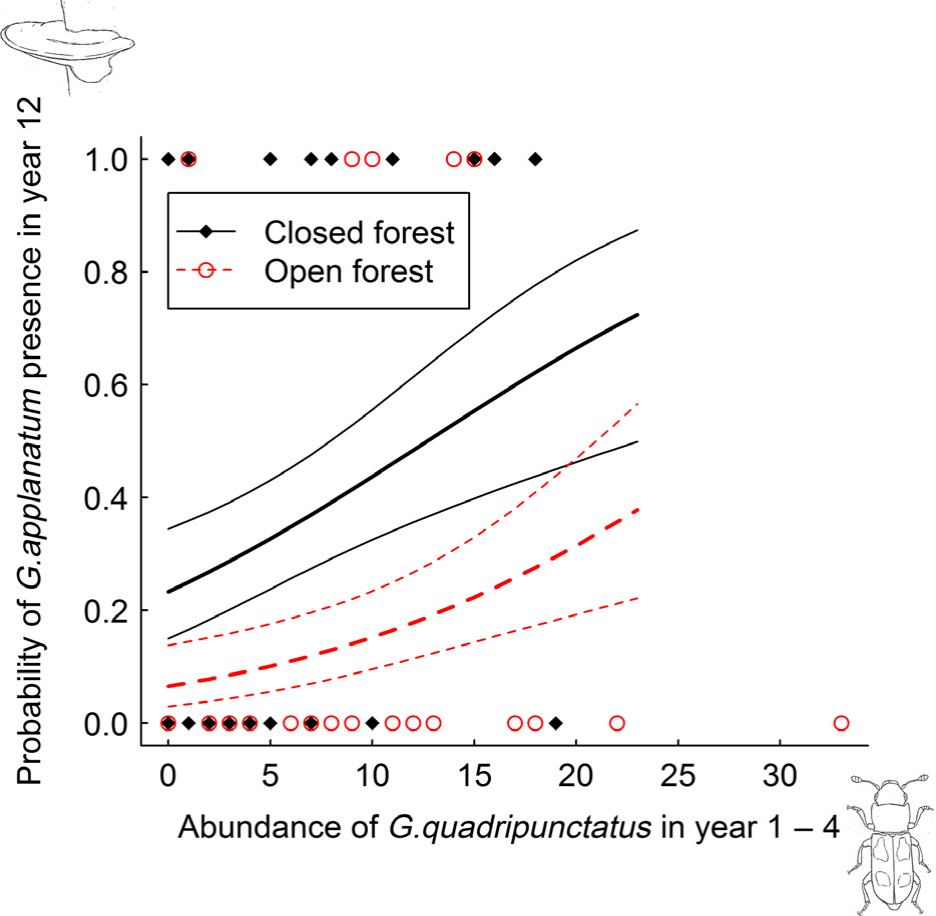
Observed presence of *G. applanatum* in year 12, with prediction lines and 95% confidence intervals based on the binomial GLM with abundance of *G. quadripunctatus* in the first 4 years after tree death as explanatory variable (Table 1). Prediction lines only extend to 23 individuals of *G. quadripunctatus*.

**Figure 3:**
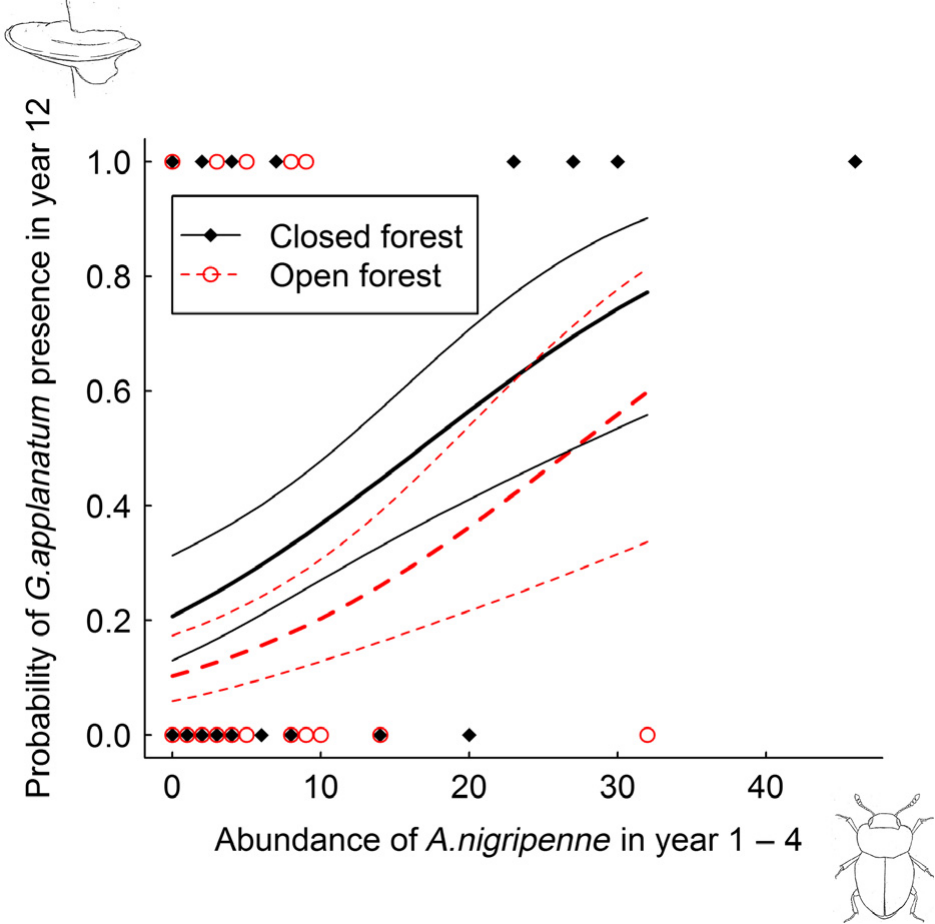
Observed presence of *G. applanatum* in year 12, with prediction lines and 95% confidence intervals based on the binomial GLM with abundance of *A. nigripenne* in year 1–4 after tree death as explanatory variable (Table 1). Prediction lines only extend to 32 individuals of *A. nigripenne*.

Of the three species of fungi selected for analysis, only the saprotrophic polypore *G. applanatum* was affected by abundance of fungivores in the first 4 years after tree death. *G. applanatum* had a positive response to abundance of fungivorous sap beetles (Nitidulidae, *P* = 0.06) and round fungus beetles (Leiodidae, *P* = 0.04) (Table S3). The most abundant sap beetles were *Glischrochilus hortensis* (Geoffroy, 1785) (58% of the Nitidulidae individuals) and *G. quadripunctatus* (Linnaeus, 1758) (42%), and the most abundant round fungus beetles were *Agathidium nigripenne* (Fabricius, 1792) (51% of the Leiodidae individuals) and *Anisotoma humeralis* (Fabricius, 1792) (14%). Analyzing these four species separately showed that the polypore *G. applanatum* was more likely to be present 12 years after tree death at sites where the fungivorous beetles *G. quadripunctatus* (*P* = 0.07) and *A. nigripenne* (*P* = 0.03) had been abundant during the first 4 years after tree death, than on sites without this colonization history (Table 1, Figs 2 and 3).

**Table 1:** Presence of the basidiomycete *G. applanatum* in year 12 after tree death explained by abundance of the fungivores *G. quadripunctatus, G. hortensis, A. humeralis,* or *A. nigripenne* in year 1–4 after three death, habitat type (open/closed forest), and site coordinates in a generalized linear model (binomial distribution and logit link). *n* = 55

	Estimate	Standard error	*z*‐value	*P*‐value
Intercept	−710.30	602.60	−1.18	0.239
Fungivorous beetle *G. quadripunctatus*	0.09	0.05	1.81	0.071
Habitat (Open forest)	−1.46	0.74	−1.97	0.048
*x* coordinate	1.1 × 10^−4^	7.7 × 10^−5^	1.36	0.173
*y* coordinate	9.7 × 10^−5^	8.4 × 10^−5^	1.16	0.248
Null deviance: 62.40 on 54 degrees of freedom Residual deviance: 55.44 on 50 degrees of freedom				
Intercept	−609.10	602.50	−1.01	0.312
Fungivorous beetle *G. hortensis*	0.04	0.03	1.31	0.191
Habitat (Open forest)	−1.34	0.73	−1.83	0.068
*x* coordinate	8.9 × 10^−5^	7.6 × 10^−5^	1.17	0.242
*y* coordinate	8.4 × 10^−5^	8.4 × 10^−5^	0.99	0.321
Null deviance: 62.40 on 54 degrees of freedom Residual deviance: 57.12 on 50 degrees of freedom				
Intercept	−792.40	622.50	−1.27	0.203
Fungivorous beetle *A. nigripenne*	0.08	0.04	2.13	0.034
Habitat (Open forest)	−0.83	0.70	−1.18	0.238
*x* coordinate	1.1 × 10^−4^	7.9 × 10^−5^	1.40	0.163
*y* coordinate	1.1 × 10^−4^	8.7 × 10^−5^	1.26	0.210
Null deviance: 62.40 on 54 degrees of freedom Residual deviance: 53.37 on 50 degrees of freedom				
Intercept	−570.90	588.90	−0.97	0.332
Fungivorous beetle *A. humeralis*	−0.01	0.14	−0.09	0.930
Habitat (Open forest)	−0.99	0.71	−1.40	0.160
*x* coordinate	8.2 × 10^−5^	7.3 × 10^−5^	1.12	0.264
*y* coordinate	7.8 × 10^−5^	8.2 × 10^−5^	0.95	0.341
Null deviance: 62.40 on 54 degrees of freedom Residual deviance: 58.79 on 50 degrees of freedom				

Although not strictly significant, an increase in abundance of the fungivorous sap beetle *G. quadripunctatus* from 0 to 20 individuals in early succession increased probability of *G. applanatum* presence in late succession with as much as 43% in closed and 25% in open habitat (Fig. 2), as predicted from the model (Table 1). Increase in abundance of the round fungus beetle *A. nigripenne* from 0 to 20 individuals in early succession was predicted to increase probability of *G. applanatum* presence in late succession with about 35% in closed and 25% in open habitat (Fig. 3). Thus, although *P*‐values were not very small, the effect sizes of the fungivores were noticeable.

The abundance of the fungivorous beetles *G. quadripunctatus* and *A. nigripenne* in year 1–4 was not correlated with any of the wood‐decay fungi that were registered in year 4 (Table S4). Thus, these fungivores did not seem to be attracted to or hatching from any of the fungal fruiting bodies present in year 4 after tree death.

### Effect of early wood‐boring beetles on late successional fungi

**Figure 4:**
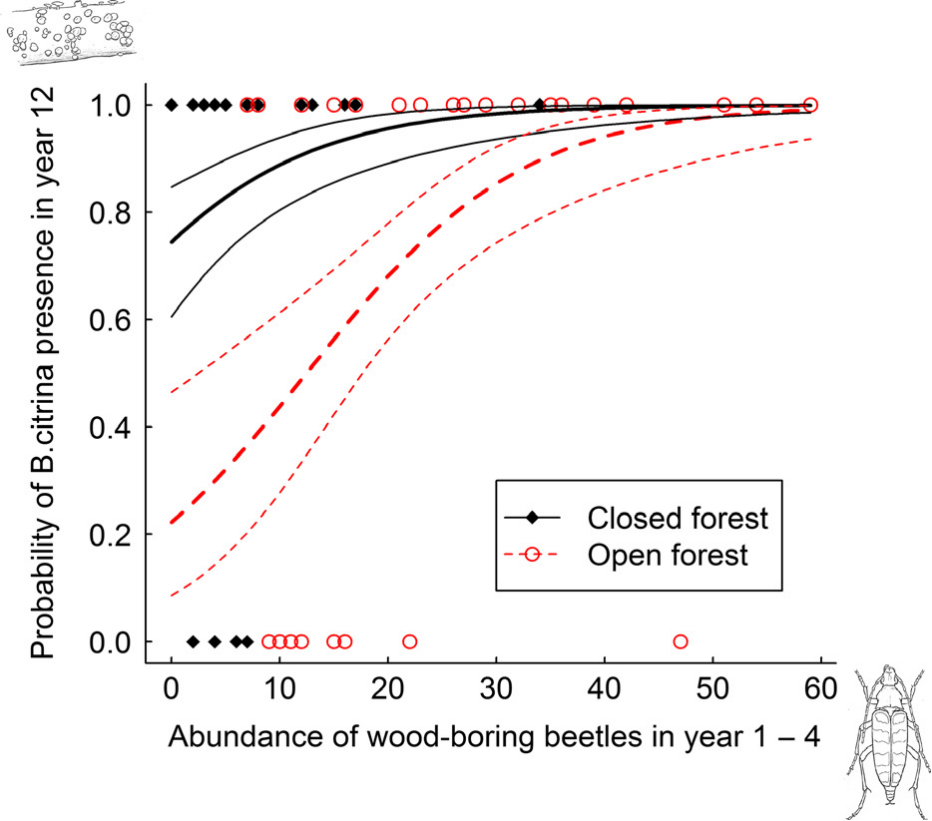
Observed presence of *B. citrina* in year 12, with prediction lines and 95% confidence intervals based the on binomial GLM (logit link) with abundance of wood‐boring beetles in the first 4 years after tree death as explanatory variable (Table 2).

Of the three species of fungi selected for analysis, only the ascomycete *B. citrina* was more likely to be present in year 12 after tree death at sites where wood‐boring beetles had been abundant during the first 4 years after tree death (Table 2, Fig. 4).

**Table 2:** Presence of the ascomycete *B. citrina* in year 12 after tree death explained by abundance of wood‐boring beetles in year 1–4, habitat type (open/closed forest) and site coordinates in a generalized linear model (binomial distribution and logit link). *n* = 55

	Estimate	Standard error	*z*‐value	*P*‐value
Intercept	−2164.00	864.30	−2.50	0.012
Wood‐boring beetles	0.10	0.05	2.04	0.042
Habitat (Open forest)	−2.32	1.10	−2.12	0.034
*x* coordinate	2.4 × 10^−4^	9.5 × 10^−5^	2.51	0.012
*y* coordinate	3.0 × 10^−4^	1.2 × 10^−4^	2.50	0.013
Null deviance: 62.40 on 54 degrees of freedom Residual deviance: 47.28 on 50 degrees of freedom				

An increase in abundance of wood‐boring beetles from 0 to 20 individuals in early succession was predicted to increase the probability of *B. citrina* presence with about 13% in closed and 45% in open forest (Fig. 4).

**Figure 5:**
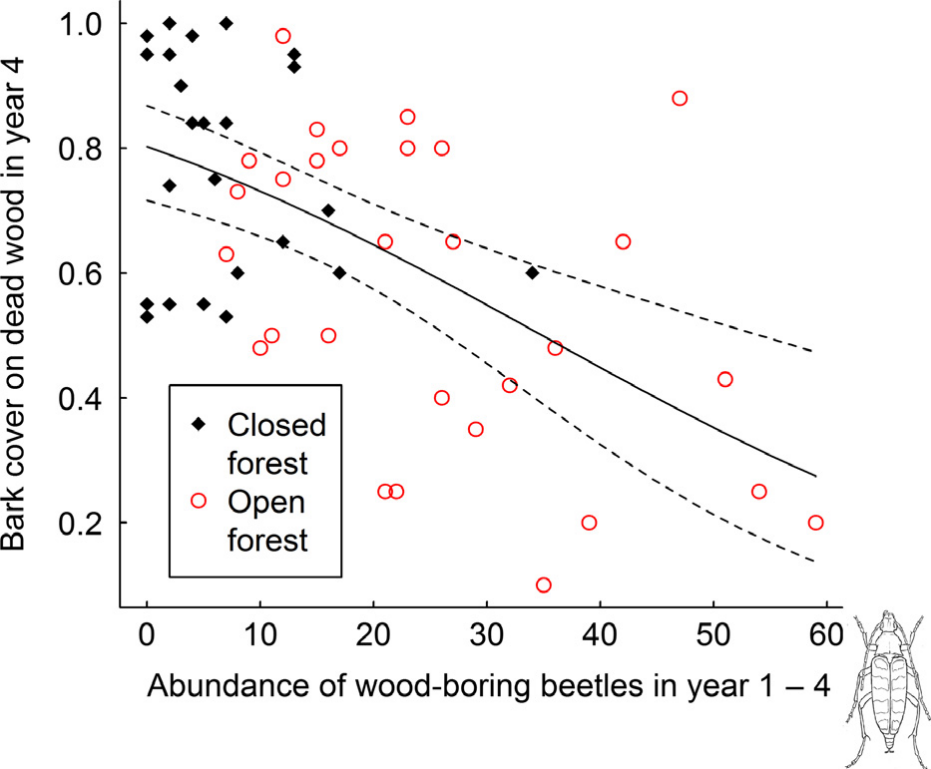
Bark cover (0–1, 1 = 100% cover) remaining in year 4 after tree death explained by abundance of wood‐boring beetles during the first 4 years after tree death. Prediction line with 95% confidence intervals from binomial GLM (logit link) explaining bark cover by abundance of wood‐boring beetles (estimate = −0.04 ± 0.02 standard error, *z*‐value = −2.01, *P*‐value = 0.045, *n* = 55).

The aspen high stumps and logs had lost significantly more bark in year 4 at sites where wood‐boring beetles had been abundant during the first 4 years after tree death (Fig. 5).

Although bark cover in year 4 did not affect *B. citrina* in year 12 if dead wood object (high stump/log) remained in the model, a negative interaction was found if dead wood object was excluded (Table 3). This was expected as the two variables were clearly correlated. Bark cover was significantly lower on logs than high stumps in early stages of decay (mean bark cover in year 4; logs = 45%, high stumps = 86%, Wilcoxon rank‐sum test; *W* = 423.50, *P*‐value <0.001), and *Bisporella citrina* occurred more often on logs than on high stumps in year 12 (Table 3).

**Table 3:** Presence of *B. citrina* in year 12 after tree death explained by bark cover (0–1, 1 = 100% cover) in year 4, dead wood object (high stump/log), and site coordinates in generalized linear models (binomial distribution and logit link). *n* = 106

	Estimate	Standard error	*z*‐value	*P*‐value
Intercept	−1679.00	655.70	−2.56	0.010
Bark cover	0.98	1.15	0.86	0.392
Object (high stump)	−4.77	1.02	−4.66	<0.001
*x* coordinate	2.2 × 10^−4^	8.5 × 10^−5^	2.63	0.009
*y* coordinate	2.3 × 10^−4^	9.1 × 10^−5^	2.55	0.011
Null deviance: 139.46 on 105 degrees of freedom Residual deviance: 73.79 on 101 degrees of freedom				
Intercept	−416.40	428.00	−0.97	0.331
Bark cover	−0.03	0.07	−3.89	<0.001
*x* coordinate	3.8 × 10^−5^	5.4 × 10^−5^	0.70	0.482
*y* coordinate	5.9 × 10^−5^	6.0 × 10^−5^	0.99	0.321
Null deviance: 139.46 on 105 degrees of freedom Residual deviance: 117.23 on 102 degrees of freedom				

## Discussion

Our results strongly suggest that the establishment of fungi is affected by the colonization history of beetles in early succession of dead wood and that this priority effect is evident in two habitats with presumably quite different microclimates; closed and open forest. The predicted presence of the wood‐decay fungi increased with as much as 13–45% with increasing fungivore or wood‐boring beetle abundance. The only fungus species with no response to insect colonization history was parasitic and could have infected the trees prior to tree death.

Our and previous research show that priority effects are important in heterotrophic communities (Shorrocks and Bingley 1994; Fukami et al. 2010; Weslien et al. 2011; Dickie et al. 2012; Ottosson et al. 2014). Comparisons of the strength of priority effects in different ecosystems are largely lacking but Chase (2010) showed that productivity mediates the strength of priority effects in autotrophic ecosystems. While productivity might not be directly applicable to heterotrophic communities, patch size and patch continuity may have similar effects. Patch continuity is very long in the dead wood system and both our correlative field study and that of Weslien et al. (2011) indicate that priority effects from early colonizing wood‐living beetles have a long‐lasting and strong influence on the establishment of wood‐decay fungi. Experimental or comparative studies including a wide range of short‐lived and long‐lived habitats are needed to establish whether priority effects in heterotrophic communities are modulated by patch continuity.

Strong priority effects can increase beta‐diversity by leading to different species assemblages in similar habitats (Chase 2010). The dead wood community in boreal forests is remarkably species rich, with about 25% of all forest species associated with dead wood (Stokland et al. 2012). The priority effects in dead wood communities found in our and previous studies (Fukami et al. 2010; Weslien et al. 2011; Dickie et al. 2012; Ottosson et al. 2014) might contribute to this high biodiversity by increasing beta‐diversity between habitat patches. If this is indeed the case, loss of early succession species would mean not only loss of the species themselves, but also of their priority effects and the subsequent successional pathways they might initiate. For instance, Weslien et al. (2011) found that colonization by the wood‐boring beetle *Hylurgops palliatus* (Gyllenhal, 1813) in early succession had an indirect positive effect on the endangered beetle *Peltis grossa* (Linnaeus, 1758) in late succession of spruce dead wood. Thus, loss of *H. palliatus* from a region would presumably have a negative influence on *P. grossa*. Similarly, our study suggests that loss or reduced abundance of the round fungus beetle *A. nigripenne* in early succession might reduce the probability that the polypore *G. applanatum* will be present in late succession of aspen dead wood. This is important, as other studies indicate that the present forest management regime might lead to profound shifts in the abundance and composition of early succession beetle communities in a long time perspective (Kouki et al. 2012; Vindstad et al. unpublished data).

The fungivorous beetles *A. nigripenne* and *G. quadripunctatus* seemed to facilitate subsequent establishment of the wood‐decay fungus *G. applanatum* and thereby follow the prediction of Connell and Slatyer (1977) that heterotrophic communities develop through facilitative succession. While Weslien et al. (2011) also found an example of inhibitory succession, all the priority effects in our study were positive. It seems that the nature of the priority effect depends on the biology of the study species. For instance, in both our study and the study of Weslien et al. (2011), the activity of wood‐boring beetles seemed to facilitate bark loss, but whereas the polypore *F. pinicola* preferred higher bark cover and therefore responded negatively to wood‐boring beetles (Weslien et al. 2011), *B. citrina* is known to prefer no bark cover (Hallingbäck and Aronsson 1998) and responded positively to wood‐boring beetles in our study.

While wood‐boring beetles can function as ecosystem engineers that alter the habitat and thereby affect species in late succession, fungivorous beetles do not impact the structure of the dead wood per se. Presumably, priority effects of fungivorous beetles in early succession on wood‐decay fungi in late succession are mediated through spore dispersal or preferential grazing. While preferential grazing can have significant short‐term effects on fungal communities (Crowther et al. 2011; A'Bear et al. 2014), this mechanism inherently facilitates one fungus species while inhibiting another. As we did not find any negative relationships between fungivores and fungi in year 4 or year 12, we consider preferential grazing to be a less likely explanation for the positive effect of fungivores. Spore dispersal seems to be the most likely mechanism in our study, and adults of both *A. nigripenne* and *G. quadripunctatus* are known to visit sporulating polypores (Hågvar and Økland 1997; Hågvar 1999; Økland 2002; Nikitsky and Schigel 2004; Schigel 2011), presumably to feed on spores.

There are certain well‐known cases of spore dispersal by insects (Ingold 1953), such as the bark beetles that act as vectors for pathogenic fungi (Webber 2004). However, apart from these specialized relationships between specific species, the role of insects as spore dispersers is unclear. Several studies have shown that wood‐living or fungivorous insects often carry large numbers of spores on their exoskeleton or in their gut (Lim 1977; Tuno 1999; Persson et al. 2009), but the effect of such incidental spore dispersal on distribution of fungi is difficult to assess. However, exclusion studies have shown that the fungal community that establishes in dead wood without insects is significantly different to the fungal community established when insects are present (Müller et al. 2002; Strid et al. 2014). In the study by Strid et al. (2014), they included a treatment with manufactured tunnels resembling those made by wood‐boring beetles, and they found that these artificial tunnels only had a marginal effect. Thus, the effect of insects on the fungal community seemed to stem from something more than physical alteration of the substrate. Spore dispersal by fungivores in early succession should lead to strong positive priority effects such as those seen for *G. applanatum* in our study, as early arrival of fungal spores would enable the fungus to capture a large area of wood, increasing its competitive advantage against fungi arriving later (Holmer and Stenlid 1993).

*Ganoderma applanatum* was the only one of the three species of fungi tested that was positively associated with fungivores, and it is also the species most likely to be dispersed by fungivores. While the parasitic *P. tremulae* does not necessarily depend on dispersal after tree death and *B. citrina* produces small, annual fruiting bodies, *G. applanatum* is a saprotrophic polypore whose perennial fruiting bodies produce remarkable numbers of spores (Ingold 1953), and several insects have been recorded to visit its fruiting bodies (Kochetova et al. 2011; Schigel 2011; Ryvarden and Melo 2014). Tuno (1999) found that *Mycodrosophila* flies caught from fruiting bodies of *G. applanatum* both dropped and excreted large numbers of viable spores, and another *Ganoderma* species produces spores that only germinate after passage through insect intestines (Lim 1977), although reduced germination rate has also been found (Kadowaki, Leschen & Beggs 2011). For insects to function as vectors for spore dispersal, they must first contract the spores, presumably by visiting a fruiting body, and then deliver the spores in viable state to a suitable substrate. This mechanism is highly contingent upon species‐specific traits, and it is therefore not surprising that the priority effects on *G. applanatum* were only found for fungivores in two of eight families tested.

It is possible that the fungivores in early succession and the fungi in late succession simply shared habitat preferences, resulting in a positive correlation. However, such indirect correlation through shared preferences offers no explanation for why *G. applanatum* was the only fungus species that responded to abundance of fungivores. Furthermore, at least with respect to the habitat types included in our design, fungi and insects exhibited opposite habitat preferences. The fungi tended to occur more often in closed forest, while both beetle guilds were more abundant in open habitats. Nevertheless, as this is an observational study, we can but suggest causal relationships. Future studies with greater control of environmental variables or of colonization history are necessary to verify the links underlying the priority effects observed in this study. Several studies of priority effects have experimentally manipulated the order of species arrival (Shorrocks and Bingley 1994; Ejrnæs et al. 2006; Kennedy et al. 2009; Chase 2010; Fukami et al. 2010; Dickie et al. 2012), which clarifies causality. On the other hand, effects that are discernible in field studies with natural colonization despite the increased variation in both colonization history and environment are more likely to be of significance for natural processes.

## Conclusions

Our study strongly indicates that colonization history of insects in early succession has a significant, long‐lasting influence on the fungal community in dead wood. Wood‐boring beetles seemed to function as ecosystem engineers, as their activity increased bark loss from the dead wood in early decay, which facilitated the ascomycete *B. citrina* several years later. Furthermore, the positive priority effects of the fungivores *A. nigripenne* and *G. quadripunctatus* on the polypore *G. applanatum* suggest that there might be a mutual dependency between some species of fungivorous insects and fungi, possibly mediated by spore dispersal. This has important implications for conservation of wood‐decay fungi, as some species might depend not only upon substrate availability, but also on facilitation by certain wood‐living insects.

## Data Accessibility

The data associated with this study have been submitted to Dryad digital repository (http://dx.doi.org/10.5061/dryad.jg2k4).

## Conflict of Interest

None declared.

## Supporting information


**Figure S1.** Box plots showing the abundance of wood‐boring and fungivorous beetles in closed and open forest habitat.

**Table S1.** Numbers of species and individuals of xylophages and fungivores sampled in 2002–2005 (year 1–4).

**Table S2.** Species inventory of all fungal fruiting bodies registered on aspen high stumps and logs in 2013 (year 12).

**Table S3.** GLM explaining presence of *G. applanatum* in year 12 by abundance of fungivores in family Nitidulidae or Leiodidae in year 1–4.

**Table S4.** Correlation between abundance of *G. quadripunctatus* or *A. nigripenne* in year 1–4 and presence of fungi in year 4 tested in GLMs.
