# Supplementary material for: Priority effects of early successional insects influence late successional fungi in dead wood
Source: Ecol Evol. 2015 Oct 12;5(21):4896–905. doi: 10.1002/ece3.1751 (PMC4662308; doi:10.1002/ece3.1751)
Supplement: Supplementary file 1 — Figure S1. Box plots showing the abundance of wood‐boring and fungivorous beetles in closed and open forest habitat. [file ECE3-5-4896-s001.pdf]

Supporting information for *Priority effects of early successional insects influence late successional fungi in dead wood* by R. Jacobsen, T. Birkemoe and A. Sverdrup-Thygeson.

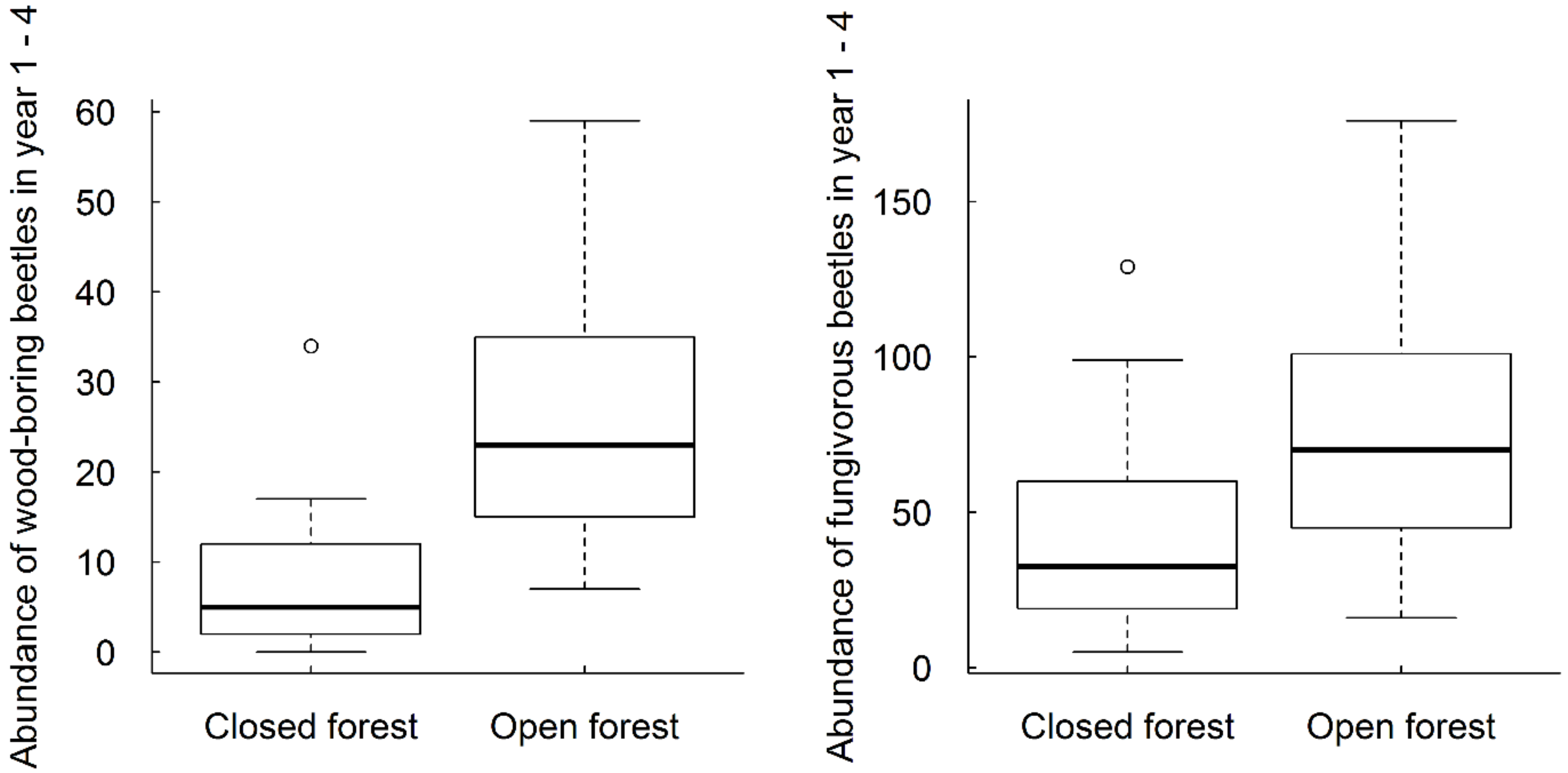

Figure S1. Box plots showing the abundance of wood-boring and fungivorous beetles in year 1 – 4 after tree death in closed and open forest habitat. Wilcoxon rank sum test p-values < 0.001 for both beetle guilds.
