# Supplementary material for: Priority effects of early successional insects influence late successional fungi in dead wood
Source: Ecol Evol. 2015 Oct 12;5(21):4896–905. doi: 10.1002/ece3.1751 (PMC4662308; doi:10.1002/ece3.1751)
Supplement: Supplementary file 2 — Table S1. Numbers of species and individuals of xylophages and fungivores sampled in 2002–2005 (year 1–4). [file ECE3-5-4896-s002.docx]

**Table S1.** Numbers of species and individuals of xylophages and

fungivores sampled in 2002 – 2005, i.e. year 1 – 4 after tree death.

| **Family** | **Species** | | **Individuals** |
| --- | --- | --- | --- |
| *Xylophages* | | | |
| Cerambycidae | | 15 | 646 |
| Curculionidae | | 7 | 236 |
| Ptinidae | | 1 | 79 |
| *Fungivores* | | | |
| Ciidae | | 14 | 209 |
| Endomychidae | | 1 | 458 |
| Erotylidae | | 4 | 260 |
| Latridiidae | | 8 | 322 |
| Leiodidae | | 11 | 773 |
| Ptinidae | | 4 | 61 |
| Staphylinidae | | 11 | 341 |
| Nitidulidae | | 3 | 1032 |
