# Supplementary material for: Priority effects of early successional insects influence late successional fungi in dead wood
Source: Ecol Evol. 2015 Oct 12;5(21):4896–905. doi: 10.1002/ece3.1751 (PMC4662308; doi:10.1002/ece3.1751)
Supplement: Supplementary file 3 — Table S2. Species inventory of all fungal fruiting bodies registered on aspen high stumps and logs in 2013 (year 12). [file ECE3-5-4896-s003.docx]

**Table S2.** Species inventory of all fungal fruiting bodies registered on aspen high stumps and logs in 2013 (year 12 after tree death), including data from three sites where only high stump or log was present (i.e. sites excluded from analysis).

| Species | No. of occurrences (58 max) | Closed forest | Open forest |
| --- | --- | --- | --- |
| *Antrodia albida* | 1 | 1 | 0 |
| *Antrodia mellita* | 1 | 1 | 0 |
| *Armillaria sp.* | 31 | 11 | 20 |
| *Artomyces pyxidatus* | 1 | 0 | 1 |
| *Ascocoryne sarcoides* | 30 | 15 | 15 |
| *Bisporella citrina* | 41 | 20 | 21 |
| *Bjerkandera adusta* | 6 | 4 | 2 |
| *Bolbitius reticulatus* | 3 | 2 | 1 |
| *Botryohypochnus isabellinus* | 1 | 0 | 1 |
| *Calocera cornea* | 13 | 7 | 6 |
| *Capitotricha bicolor* | 1 | 1 | 0 |
| *Cerrena unicolor* | 2 | 1 | 1 |
| *Cheimonophyllum candidissimum* | 1 | 1 | 0 |
| *Chlorencoelia versiformis* | 1 | 0 | 1 |
| *Chlorociboria sp.* | 9 | 4 | 5 |
| *Crepidotus lundellii* | 1 | 0 | 1 |
| *Dacrymyces sp.* | 5 | 3 | 2 |
| *Datronia mollis* | 13 | 9 | 4 |
| *Fomitopsis pinicola* | 2 | 0 | 2 |
| *Galerina marginata* | 18 | 8 | 10 |
| *Galerina sp.* | 2 | 1 | 1 |
| *Ganoderma applanatum* | 14 | 9 | 5 |
| *Gloeophyllum sepiarium* | 8 | 1 | 7 |
| *Gymnopilus penetrans* | 3 | 2 | 1 |
| *Gyromitra infula* | 4 | 1 | 3 |
| *Hymenochaete cinnamomea* | 1 | 0 | 1 |
| *Hyphodontia quercina* | 1 | 1 | 0 |
| *Hypholoma capnoides* | 5 | 2 | 3 |
| *Hypsizygus ulmarius* | 3 | 3 | 0 |
| *Inonotus radiatus* | 1 | 0 | 1 |
| *Laxitextum bicolor* | 1 | 0 | 1 |
| *Lentinellus castoreus coll.* | 4 | 3 | 1 |
| *Mollisia cinerea coll.* | 6 | 2 | 4 |
| *Mycena epipterygia* | 1 | 1 | 0 |
| *Mycena haematopus* | 1 | 0 | 1 |
| *Mycena leptocephala* | 1 | 0 | 1 |
| *Mycena metata* | 3 | 0 | 3 |
| *Mycena sp.* | 2 | 1 | 1 |
| *Nemania sp.* | 27 | 14 | 13 |
| *Oxyporus corticola* | 5 | 2 | 3 |
| *Phellinus populicola* | 3 | 1 | 2 |
| *Phellinus tremulae* | 19 | 12 | 7 |
| *Phlebia tremellosa* | 5 | 0 | 5 |
| *Pholiota limonella* | 2 | 1 | 1 |
| *Pholiota sp.* | 1 | 0 | 1 |
| *Pholiota squarrosa* | 3 | 1 | 2 |
| *Phyllotopsis nidulans* | 1 | 1 | 0 |
| *Postia hibernicus* | 1 | 0 | 1 |
| *Postia tephroleuca* | 1 | 0 | 1 |
| *Psathyrella spadicea* | 1 | 1 | 0 |
| *Pycnoporus cinnabarinus* | 1 | 0 | 1 |
| *Resupinatus poriaeformis* | 1 | 1 | 0 |
| *Schizopora paradoxa* | 2 | 0 | 2 |
| *Scutellinia sp.* | 3 | 1 | 2 |
| *Sidera vulgaris* | 1 | 0 | 1 |
| *Stereum rugosum* | 1 | 1 | 0 |
| *Trametes betulina* | 2 | 1 | 1 |
| *Trametes hirsuta* | 1 | 1 | 0 |
| *Trametes ochracea* | 45 | 25 | 20 |
| *Tubaria minutalis* | 11 | 8 | 3 |
| *Tyromyces chioneus* | 1 | 0 | 1 |
| *Xylaria hypoxylon* | 5 | 4 | 1 |
