# Supplementary material for: Priority effects of early successional insects influence late successional fungi in dead wood
Source: Ecol Evol. 2015 Oct 12;5(21):4896–905. doi: 10.1002/ece3.1751 (PMC4662308; doi:10.1002/ece3.1751)
Supplement: Supplementary file 4 — Table S3. GLM explaining presence of G. applanatum in year 12 by abundance of fungivores in family Nitidulidae or Leiodidae in year 1–4. [file ECE3-5-4896-s004.docx]

**Table S3.** Presence of the basidiomycete *G. applanatum* in year 12 after tree death explained by abundance of the fungivores in family Nitidulidae or Leiodidae in year 1 – 4, habitat type (open/closed forest) and site coordinates in a generalized linear model (binomial distribution and logit link). n = 55.

|  | **Estimate** | **SE** | **z-value** | **p-value** |
| --- | --- | --- | --- | --- |
| Intercept | - 666.80 | 616.80 | - 1.08 | 0.280 |
| Nitidulidae | 0.06 | 0.03 | 1.92 | 0.055 |
| Habitat (Open forest) | -1.69 | 0.80 | - 2.11 | 0.035 |
| x coordinate | 1.0 x 10^-4^ | 7.8 x 10^-5^ | 1.29 | 0.196 |
| y coordinate | 9.1 x 10^-5^ | 8.6 x 10^-5^ | 1.06 | 0.291 |
|  |  |  |  |  |
| *Null deviance: 62.40 on 54 degrees of freedom*  *Residual deviance: 54.80 on 50 degrees of freedom* | | | | |
|  |  |  |  |  |
| Intercept | - 908.10 | 654.50 | - 1.39 | 0.165 |
| Leiodidae | 0.09 | 0.045 | 2.06 | 0.040 |
| Habitat (Open forest) | 0.28 | 1.056 | 0.26 | 0.793 |
| x coordinate | 1.3 x 10^-4^ | 8.4 x 10^-5^ | 1.50 | 0.133 |
| y coordinate | 1.3 x 10^-4^ | 9.1 x 10^-5^ | 1.37 | 0.171 |
| Leiodidae:Habitat (Open) | - 0.10 | 0.06 | - 1.65 | 0.099 |
|  |  |  |  |  |
| *Null deviance: 62.40 on 54 degrees of freedom*  *Residual deviance: 52.99 on 49 degrees of freedom* | | | | |
