# Supplementary material for: Priority effects of early successional insects influence late successional fungi in dead wood
Source: Ecol Evol. 2015 Oct 12;5(21):4896–905. doi: 10.1002/ece3.1751 (PMC4662308; doi:10.1002/ece3.1751)
Supplement: Supplementary file 5 — Table S4. Correlation between abundance of G. quadripunctatus or A. nigripenne in year 1–4 and presence of fungi in year 4 tested in GLMs. [file ECE3-5-4896-s005.docx]

**Table S4.** Correlation between abundance of *Glischrochilus quadripunctatus* (G.quad) or *Agathidium nigripenne* (A.nig) and presence of the fungi *Schizopora paradoxa, Trametes betulina, Cerrena unicolor, Trametes hirsuta* and *Phellinus tremulae* in year 4 after tree death tested by binomial GLMs (logit link) with habitat (open or closed forest) and site coordinates (x coord and y coord) as covariates.

|  | *Schizopora paradoxa* year 4 | | | | *Trametes betulina* year 4 | | | |
| --- | --- | --- | --- | --- | --- | --- | --- | --- |
|  | **Estimate** | **Standard error** | **z-value** | **p-value** | **Estimate** | **Standard error** | **z-value** | **p-value** |
| Intercept | 864.30 | 769.00 | 1.12 | 0.261 | -642.30 | 664.50 | -0.97 | 0.333 |
| G. quad | -0.03 | 0.05 | -0.58 | 0.559 | -0.03 | 0.06 | -0.46 | 0.643 |
| Habitat (Open) | 3.24 | 1.18 | 2.76 | 0.006 | 1.46 | 0.77 | 1.91 | 0.056 |
| x coord | -1.2 x 10^-4^ | 9.1 x 10^-5^ | -1.29 | 0.196 | 9.5 x 10^-5^ | 8.4 x 10^-5^ | 1.12 | 0.264 |
| y coord | -1.2 x 10^-4^ | 1.1 x 10^-4^ | -1.11 | 0.266 | 8.8 x 10^-5^ | 9.3 x 10^-5^ | 0.95 | 0.343 |
|  |  |  |  |  |  |  |  |  |
| Intercept | 8.60 | 763.60 | 1.13 | 0.258 | -707.20 | 675.20 | -1.05 | 0.294 |
| A. nig | 0.06 | 0.05 | 1.11 | 0.266 | 0.04 | 0.04 | 1.20 | 0.230 |
| Habitat (Open) | 3.59 | 1.35 | 2.66 | 0.007 | 1.58 | 0.79 | 2.00 | 0.045 |
| x coord | -1.2 x 10^-4^ | 9.1 x 10 ^-5^ | -1.32 | 0.188 | 1.0 x 10^-4^ | 8.5 x 10^-4^ | 1.19 | 0.234 |
| y coord | -1.2 x 10^-4^ | 1.2 x 10^-4^ | -1.12 | 0.263 | 9.7 x 10^-5^ | 9.4 x 10^-5^ | 1.03 | 0.304 |
|  | *Cerrena unicolor* year 4 | | | | *Trametes hirsuta* year 4 | | | |
|  | **Estimate** | **Standard error** | **z-value** | **p-value** | **Estimate** | **Standard error** | **z-value** | **p-value** |
| Intercept | -204.80 | 895.30 | -0.23 | 0.819 | -477.80 | 689.40 | -0.69 | 0.488 |
| G. quad | -0.07 | 0.07 | -0.93 | 0.351 | 0.01 | 0.05 | 0.35 | 0.728 |
| Habitat (Open) | 0.79 | 0.80 | 0.99 | 0.321 | 2.90 | 1.12 | 2.59 | 0.009 |
| x coord | 1.0 x 10^-4^ | 1.1 x 10^-4^ | 0.91 | 0.362 | 2.5 x 10^-5^ | 8.3 x 10^-5^ | 0.31 | 0.760 |
| y coord | 2.1 x 10^-5^ | 1.3 x 10^-4^ | 0.17 | 0.865 | 6.9 x 10^-5^ | 9.7 x 10^-5^ | 0.71 | 0.474 |
|  |  |  |  |  |  |  |  |  |
| Intercept | -218.30 | 877.30 | -0.25 | 0.804 | -428.50 | 687.00 | -0.62 | 0.532 |
| A. nig | 0.01 | 0.04 | 0.02 | 0.986 | 0.04 | 0.05 | 0.75 | 0.453 |
| Habitat (Open) | 0.56 | 0.75 | 0.74 | 0.460 | 3.18 | 1.18 | 2.69 | 0.007 |
| x coord | 1.1 x 10^-4^ | 1.1 x 10^-4^ | 0.95 | 0.344 | 1.6 x 10^-5^ | 8.2 x 10^-5^ | 0.19 | 0.848 |
| y coord | 2.3 x 10^-5^ | 1.2 x 10^-4^ | 0.19 | 0.850 | 6.3 x 10^-5^ | 9.7 x 10^-5^ | 0.65 | 0.517 |
|  | *Phellinus tremulae* year 4 | | | |  |  |  |  |
|  | **Estimate** | **Standard error** | **z-value** | **p-value** |  |  |  |  |
| Intercept | -5.95 | 521.10 | -0.01 | 0.991 |  |  |  |  |
| G. quad | 0.018 | 0.031 | 0.59 | 0.552 |  |  |  |  |
| Habitat (Open) | -0.46 | 0.58 | -0.79 | 0.428 |  |  |  |  |
| x coord | 2.4 x 10^-5^ | 6.3 x 10^-5^ | 0.39 | 0.698 |  |  |  |  |
| y coord | -1.3 x 10^-6^ | 7.3 x 10^-5^ | -0.02 | 0.985 |  |  |  |  |
|  |  |  |  |  |  |  |  |  |
| Intercept | 29.22 | 520.10 | 0.06 | 0.955 |  |  |  |  |
| A. nig | -0.01 | 0.05 | -0.32 | 0.751 |  |  |  |  |
| Habitat (Open) | -0.47 | 0.59 | -0.80 | 0.425 |  |  |  |  |
| x coord | 2.0 x 10^-5^ | 6.3 x 10^-5^ | 0.31 | 0.756 |  |  |  |  |
| y coord | -6.2 x 10^-6^ | 7.3 x 10^-5^ | -0.08 | 0.933 |  |  |  |  |
